# Dielectric and electromagnetic interference shielding properties of high entropy (Zn,Fe,Ni,Mg,Cd)Fe_2_O_4_ ferrite

**DOI:** 10.1038/s41598-019-56586-6

**Published:** 2019-12-27

**Authors:** Adrian Radoń, Łukasz Hawełek, Dariusz Łukowiec, Jerzy Kubacki, Patryk Włodarczyk

**Affiliations:** 1 Łukasiewicz Research Network - Institute of Non-Ferrous Metals, Sowinskiego 5 St., 44-100 Gliwice, Poland; 2 Faculty of Mechanical Engineering, Silesian University of Technology, Konarskiego 18A St., 44-100 Gliwice, Poland; 3 A. Chelkowski Institute of Physics, University of Silesia, 75 Pułku Piechoty 1 St., 41-500 Chorzów, Poland; 4 Silesian Center for Education and Interdisciplinary Research, 75 Pułku Piechoty 1A St., 41-500 Chorzów, Poland

**Keywords:** Electrical and electronic engineering, Electronic properties and materials

## Abstract

The new (Zn,Mg,Ni,Fe,Cd)Fe_2_O_4_ high entropy ferrite with average crystallite size 11.8 nm was synthesized in two stages by annealing of co-precipitated amorphous precursor. The dielectric spectroscopy confirms, that the electrical conductivity and polarization processes are associated with the mobility of electrons in the structure of ferrite. It was concluded, that the both, high frequency complex dielectric permittivity as well as complex magnetic permeability are strongly temperature and frequency dependent. The AC electrical conductivity is associated with quantum mechanical tunneling of electrons and related to the transfer of charge carriers between Fe^2+^ and Fe^3+^ ions. Moreover, the microwave absorption properties were determined. The best microwave absorption properties have been confirmed in the frequency range 1.9 to 2.1 GHz for a layer which is 0.8–1 cm thick. For this range, reflection loss (RL) is lower than −25 dB and shielding effectiveness (SE) lower than −50 dB.

## Introduction

High entropy oxides (HEOs) are a new group of materials developed in 2015^1^. Recently, HEOs were tested in different applications, especially in electronics according to their dielectric properties. Sekar *et al*. has pointed out, that the HEOs can be used for reversible energy storage^2^. According to the studies of Berardan *et al*., there is possibility of insertion of Li-ions in the structure of HEOs, allowing to obtain material with high ionic conductivity i.e. approximately 10^−3^ S cm^−1 ^^3^. The rare-earth-based HEOs can be used as absorbers of light from the entire visible spectrum. The optical properties are closely related to the presence of multivalent praseodymium, whereas crystal structure and phase purity is controlled by cerium. Moreover, the influence of chemical composition and entropy on magnetic properties was recently described^4^. Different types of HEOs such as transition-metal-based HEOs, rare-earth-based HEOs and mixed HEOs were synthesized and tested until now. The stabilization of entropy in oxides leads to the formation of single-phase structures within 5 or more cations in equiatomic concentration. Several compositions and elemental combinations allowed to synthesize HEOs with different crystal structures, such as rocksalt, fluorite and perovskite^1,5,6^.

The ferrites belong to group of inorganic materials with spinel or inverse spinel structure. The cation distribution in a unit cell of spinel can be generally expressed by the formula: (Me^2+^_1-γ_Fe^3+^_γ_)_8_[Me^2+^_γ_Fe^3+^_2-γ_]_8_O^2−^_32_. The magnetite crystalizes in inverse cubic spinel structure, in which Fe^3+^ ions occupy tetrahedral and octahedral sites, whereas Fe^2+^ ions only octahedral sites. The introduction of different Me^2+^ and Me^3+^ ions in the structure of magnetite is well known and described in the literature. Moreover, the introduced Me^2+^ ions can occupy tetrahedral and/or octahedral sites unlike the Fe^2+^ ions^7,8^. This introduces the disorder into the structure, which in consequence leads to the altered magnetic and dielectric properties of ferrites^9–12^. These properties are relevant to the application of ferrites in microwave industry, magnetic recording, catalysts, sensors, drug delivery and magnetic resonance imaging^13–16^. Many of ferrites nanoparticles and hybrid systems with metal nanoparticles were recently developed and tested as biomarkers, magneto-plasmonic materials and catalysts^17–19^. The introduction of different chemical elements in the spinel structure changes magnetic, optical and electric properties. According to that, the application of them also changes. For example, R. Sharma *et al*. tested the influence of the introduction of different ions into magnetite structure on the photocatalytic activity in the degradation of organic pollutants. They confirmed, that the introduction of Cu^2+^ ions increases significantly rate of degradation reaction in comparison with CoFe_2_O_4_ NPs^20^. Moreover, it was confirmed, that the addition of cobalt and other ions such as Zn^2+^, Mn^2+^ and Ni^2+^ changes the magnetic properties of ferrites such as anisotropy, coercivity, Curie temperature, and saturation magnetization. The modulation of these properties can allow synthesizing materials, which can be used in a wide range of medical applications such as drug delivery, biosensors, magnetic hyperthermia and magnetic resonance imaging^21^. Interestingly, some of the introduced ions can react with other nanoparticles deposited on the surface of ferrites. For example, the synthesis of gold nanoparticles on the surface of CuFe_2_O_4_ nanoparticles can results not only in formation of Au/CuFe_2_O_4_ but also with alloying of Au by Cu^19^. According to that, the introduction of different ions simultaneously can result in formation of nanoparticles with unique multifunctional properties.

The addition of different chemical elements changes the electric, dielectric and microwave absorption properties of ferrites. For example, doping cobalt ferrites (CoFe_2_O_4_) by Cr^3+^ ions caused the increase of resistivity of material^22^. The microwave absorption properties of ferrites are associated with synergetic effect of magnetic and dielectric loss. The influence of the chemical composition of (Ni_1−*x*−*y*_Co_*x*_Zn_*y*_)Fe_2_O_4_ spinel ferrites on the electromagnetic interference (EMI) shielding properties was described by Xie *et al*.^23^. They reported, that the best microwave absorber at 8.64–11.2 GHz frequency band is the (Ni_0.407_Co_0.207_Zn_0.386_)Fe_2_O_4_ and absorbing frequency band is strongly related to the chemical composition of ferrites.

The possibility of synthesis of high entropy oxide with spinel structure was previously confirmed by Dąbrowa *et al*.^24^. The authors have reported that the (Co,Cr,Fe,Mn,Ni)_3_O_4_ oxide with *Fd-3m* single phase can be synthesized by sintering of compressed sample prepared from milled oxides at 1050 °C by 20 h.

The aim of this study was to develop and study high entropy ferrite (HEF) in which entropy is being stabilized in tetrahedral and octahedral sites by only Me^2+^ ions. High entropy oxide was synthesized using a low-temperature two-step method. According to our knowledge, such method was not reported earlier to synthesis of HEOx. Dielectric measurements of (Zn,Mg,Ni,Fe,Cd)Fe_2_O_4_ high entropy ferrite allowed us to determine its electrical conduction mechanism and microwave absorption properties. Moreover, in the literature many results indicate that the different nanoparticles and nanocomposites can be used as EMI shielding materials in GHz region^25–28^. However, the results are presented usually for very high frequencies (>10 GHz) and at room temperature. For example, it was confirmed, that the Mn_0.7_Zn_0.3_Fe_2_O_4_ and Ni_0.7_Zn_0.3_Fe_2_O_4_ nanoparticles have very good absorption properties around 10.4 GHz^29^. In present study EMI shielding properties were examined in RF frequency range from 100 MHz to 2.1 GHz and in broad temperature range (233–343 K) in order to determine possibility of using the (Zn,Mg,Ni,Fe,Cd)Fe_2_O_4_ high entropy ferrite as radio-frequency shielding agent.

## Materials and Methods

### Synthesis of (Zn,Mg,Ni,Fe,Cd)Fe_2_O_4_ high entropy ferrite

The (Zn,Mg,Ni,Fe,Cd)Fe_2_O_4_ high entropy ferrite (HEF) was synthesized in two-step process. Firstly, the amorphous precursor was synthesized by simple co-precipitation method. For this purpose 40 mmol of FeCl_3_ and 4 mmol of FeSO_4_·7 H_2_O, ZnSO_4_·7 H_2_O, NiSO_4_·7 H_2_O, Mg(NO_3_)_2_·6 H_2_O and Cd(NO_3_)_2_·4 H_2_O of salts were added into 100 ml of deionized (DI) water. In a separate beaker, 100 ml of 2 M NaOH water solution was prepared. Afterwards, under continuous stirring NaOH solution was added to the salt solution. Finally, additional 100 ml of DI water was added to the sample and the precipitate was then aged by 72 h at ambient temperature. The precipitate was filtered using hardened quantitative filter paper and washed by DI water, ethanol and acetone. The powder was dried at 333 K. The amorphous precursor was then annealed at 793 K by 2 h.

### Materials characterization

The amorphous state of synthesized precursor as well as crystal structure of the (Zn,Mg,Ni,Fe,Cd)Fe_2_O_4_ HEF were verified using X-ray diffraction method (XRD). The powder XRD patterns were recorded at ambient temperature using X-ray diffractometer Rigaku MiniFlex 600 with a copper tube Cu Kα (*λ* = 0.15406 nm), a tube voltage of 40 kV, and a current of 15 mA, using a D/teX Ultra silicon strip detector. The crystal structure of synthesized HEF was analysed and displayed using the Rigaku PDXL software suite. Energy-dispersive X-ray (EDX) spectra, EDX maps, electron microscope images and selected area electron diffraction (SAED) patterns were obtained using a transmission electron microscope S/TEM TITAN 80–300. The images were collected in scanning transmission electron microscopy (STEM) and transmission electron microscopy (TEM) modes. The sample was prepared by redispersion of HEF in ethanol and placed on the lacey carbon films on copper TEM grid (200 mesh). The electronic structure of the sample was examined by X-ray photoelectron spectroscopy method (XPS) with used to Prevac/VGScienta R3000 photoelectron spectrometer. Monochromatic AlKα x-ray radiation (hν = 1486.6 eV) was used to obtain the photoelectron spectra of O1s, C1s, Fe2p, Cd3d, Zn2p, Ni2p_3/2_ and Mg1s of the core levels with 100 Pass Energy and 0.100 step of parameters. All photoelectron spectra were calibrated against the peaks of Au 4f_7/2_ at 83.98 eV, Ag 3d_5/2_ at 368.27 eV and Cu 2p_3/2_ at 932.67 eV of binding energy. The positions of measured photoemission lines were calibrated to C1s binding energy at 285 eV. The shape of the photoelectron lines was analyzed using the Multipak program (PHI Multipak SoftwareTM, Version 9.8.0.19) from Physical Electronics. Dielectric and magnetic permeability measurements were performed using the Concept 81 dielectric spectrometer, equipped with an Alpha and Keysight 4991E analysers and the Novo-cool temperature control system. Complex dielectric permittivity was measured for compressed samples with a diameter of 10 mm and thickness of 1.29 mm (compression pressure was equal to 30 bar) in broad frequency range from 5•10^−2^ to 1*10^6^ Hz using Alpha analyzer and in high frequency range (1*10^8^–2.1*10^9^ Hz) using Keysight analyzer. Low frequency measurements (5*10^−2^–1*10^6^ Hz) were performed in broad temperature range 173–363 K (ΔT = 10 K) in order to determine dielectric properties and electric conduction mechanism. High frequency measurements (1*10^8^–2.1*10^9^ Hz) were performed in temperature range from 233 to 343 K (ΔT = 5 K) in order to determine high frequency dielectric properties and EMI shielding properties. High frequency complex permeability was measured in the same frequency and temperature range for toroidal samples with outer diameter of 20.1 mm, inner diameter of 6.63 mm and ring height of 1.76 mm prepared by compression with *p* = 30 bar.

## Results and Discussion

**Figure 1:**
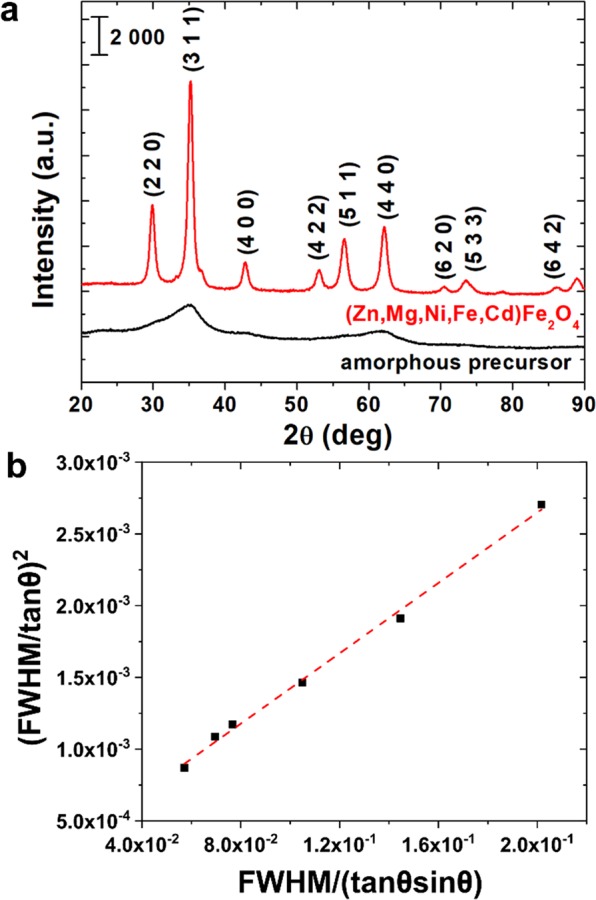
(**a**) XRD diffraction patterns of amorphous precursor and crystalline (Zn,Mg,Ni,Fe,Cd)Fe_2_O_4_ high entropy ferrite, **(b)** Halder-Wagner plot.

The XRD patterns presented in Fig. 1a confirm, that the fully amorphous precursor can be obtained using proposed co-precipitation method. After annealing by 2 hours at 793 K the crystallization of amorphous phase in spinel structure occurs. Diffraction peaks that have been identified and marked in Fig. 1a can be well described by Cd_0.1_ Fe_2_Ni_0.5_O_4_Zn_0.4_ ferrite structure (space group: 227:*Fd-3m*, DB card number: 9009921) with the lattice parameters *a* = *b* = *c* = 0.845 nm and *α* = *β* = *γ* = 90°. The average crystallite size was calculated on the basis of Halder-Wagner method, in which broadening of diffraction peaks is related to the ultrafine size of crystallites and also to the microstrain in their structure^30^. The crystallite size (*D*) and strain (*ε*) are related to the full-width at half maximum (*FWHM*) according to the equations^31^:

1 $${(\frac{{\beta }^{\ast }}{d})}^{2}=\frac{1}{D}\frac{{\beta }^{\ast }}{{d}^{2}}+{(\frac{\varepsilon }{2})}^{2}$$

2 $${\beta }^{\ast }=\frac{(FWHM)cos\theta }{\lambda }$$

3 $$d=\frac{2sin\theta }{\lambda }$$

where: *λ* is the wavelength of X-ray and *θ* is the diffraction angle. On the assumption that the Lorentzian and Gaussian components of β* are due to the size and strain, Eq. (3) can be rewritten as:

4 $${(\frac{FWHM}{tan\theta })}^{2}=\frac{K\lambda }{D}\frac{FWHM}{tan\theta sin\theta }+{(\frac{\varepsilon }{2})}^{2}$$

where *K* is a constant, herein equal to 0.94.

On the basis of analysis of slope and intercept of *(FWHM/tanθ)*^2^ vs *FWHM/(tanθsinθ)* plot (Fig. 1b) the crystallite size and strain can be determined. It was calculated, that the average crystallite size is equal to 11.8 nm and microstrain equals 0.35%.

**Figure 2:**
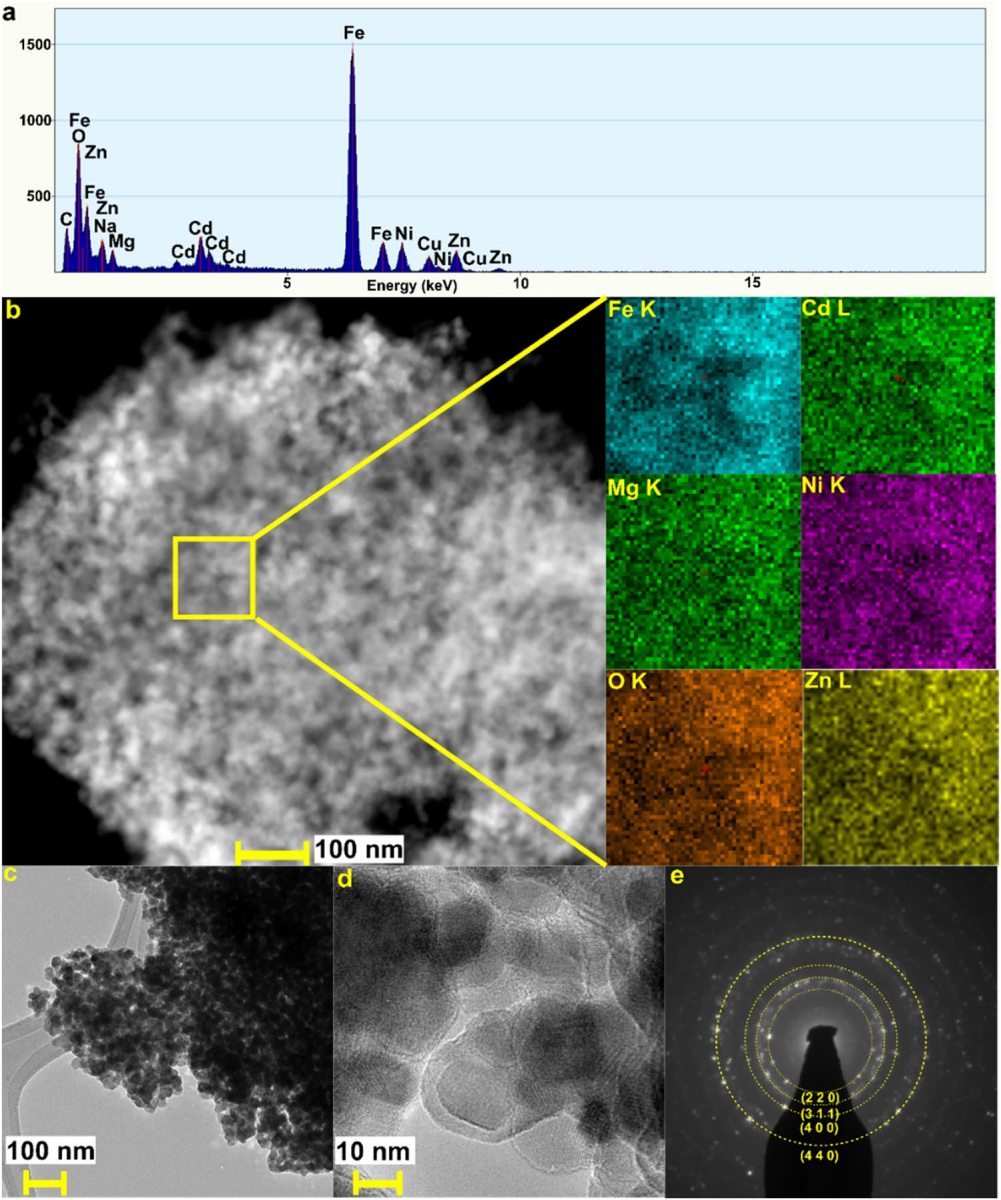
Analysis of the structure and morphology of (Zn,Mg,Ni,Fe,Cd)Fe_2_O_4_ high entropy ferrite: **(a)** EDX spectra, **(b)** STEM image with marked area and corresponding 2D STEM-EDX maps, **(c)** LRTEM image, **(d)** HRTEM image confirming nanocrystalline structure of HEF, **(e)** SAED pattern indexed with the lattice planes.

The presence of Zn, Mg, Ni, Fe and Cd in the structure of HEF was confirmed by analysis of EDX spectrum recorded at STEM mode for prepared sample. The results presented in Fig. 2a confirms, that the all introduced chemical elements are present in the structure of synthesized ferrite. Moreover, 2D STEM-EDX maps presented in Fig. 2b clearly confirm, that Fe, Cd, Mg, Ni, Zn and O are randomly distributed in the structure of ferrite. This random distribution results in the high entropy ferrite being formed after short annealing of amorphous precursor. The configurational entropy of the (Zn,Mg,Ni,Fe,Cd)Fe_2_O_4_ high entropy ferrite calculated according to the Eq. (5) is equal to 7.26 J/mol·K^2^.

5 $${S}_{config}=-\,R[{(\mathop{\sum }\limits_{i=1}^{N}{x}_{i}ln{x}_{i})}_{cation}+{(\mathop{\sum }\limits_{j=1}^{N}{x}_{j}ln{x}_{j})}_{anionn}]$$

where *x*_*i*_ and *x*_*j*_ are the mole fractions of ions in the cation and anion-sites, respectively. The influence of distribution of anions in this research is negligible, because the synthesized ferrite has only one type of anion O^2−^ and high value of entropy is associated only with presence of different cations in the structure.

Moreover, as can be seen in the Fig. 2b–d the synthesized high entropy ferrite forms large agglomerates composed of nanoparticles of high entropy ferrite. The performed analysis of SAED patterns clearly confirms the results obtained from the analysis of XRD patterns, that the synthesized material crystallizes in spinel structure, which is characteristic for ferrites.

**Figure 3:**
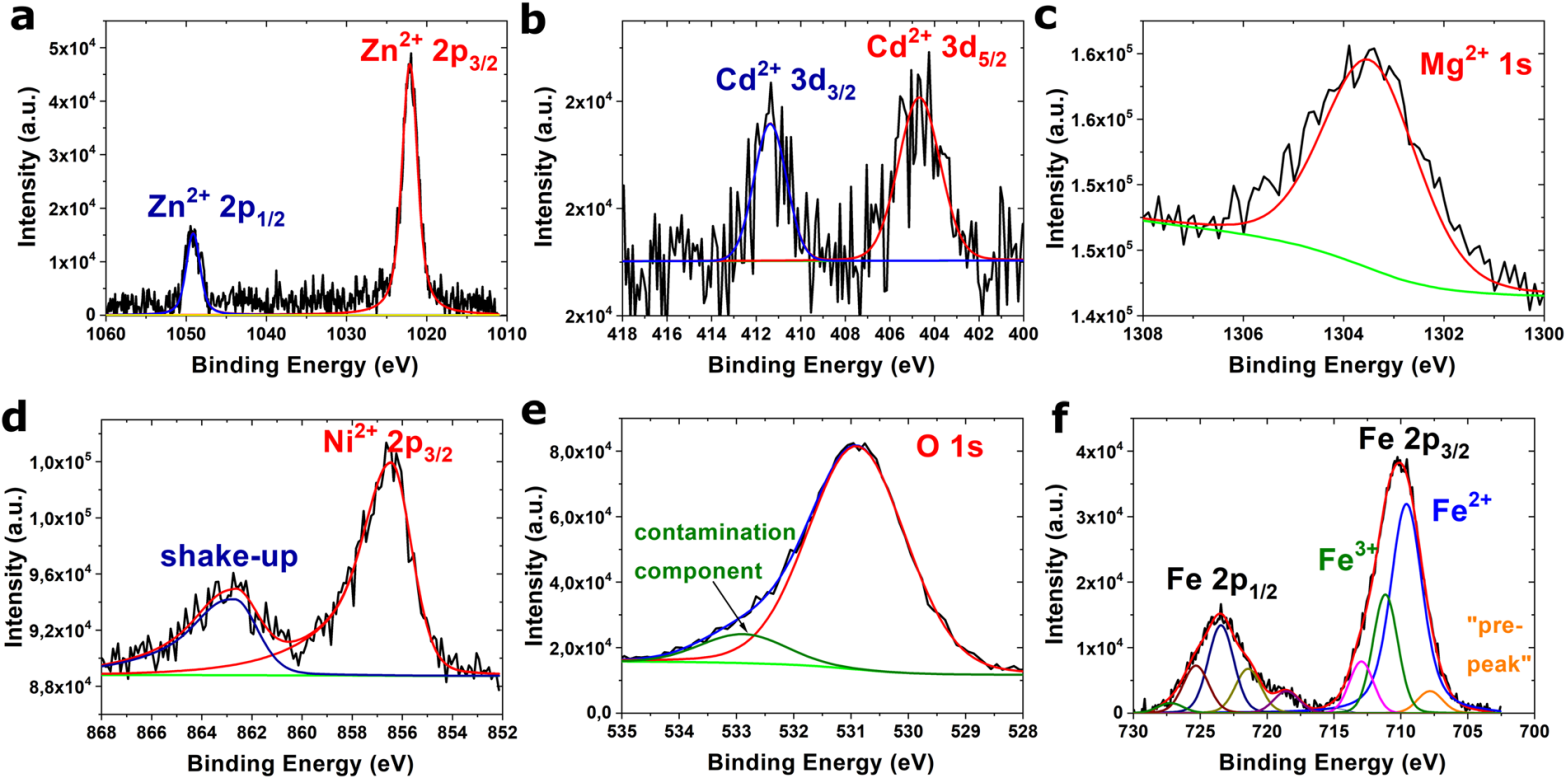
High resolution XPS spectrum of the Zn2p (**a**), Cd3d (**b**), Mg1s (**c**) and Ni2p_3/2_ (**d**) core lines; deconvoluted spectra of O1s **(e**) and Fe2p core lines (**f**). The Shirley background was subtracted in both cases.

Additionally, to confirm the presence and valence state of Zn, Mg, Ni, Fe and Cd the XPS spectra were collected for synthesized HEF. The collection of the high resolutions photoemission lines of O1s, Ni2p_3/2_, Zn2p, Mg1s, Cd3d and Fe2p were presented in Fig. 3a–d. The binding energy of the Zn2p_3/2_, Ni2p_3/2_, Mg1s and Cd3d_5/2_ peaks confirms the presence of Me^2+^ ions in the structure^32–35^. The results of deconvolution of the shape O1s and Fe2p spectra were presented in Fig. 3e,f, respectively. The O1s line consists of two components at 530.8 eV and 532.8 eV of binding energy, which can be ascribed to metal oxides and contamination, respectively. The fitting of the Fe 2p3/2 line, presented in Fig. 3f, shows a clear main contribution of two components at a binding energy of about 709.5 eV and 711 eV. Additionally, two satellite peaks, one with a small intensity and the second at a binding energy of about 712–715 eV and 719 eV can be recognised for the main peak of Fe2p multiplet. The calculations performed by Grosvenor *et al*. for high-spin Fe^3+^ and Fe^2+^ compounds indicate that the first component and satellite peak at 715 eV corresponded to contribution Fe^2+^, while second component and satellite component at 719 corresponded to contribution Fe^3+^^36^. Hence, the iron presented in the sample is in a mixed 2+/3+ valence state, which also confirms the formation of ferrite structure.

Moreover, on the basis of EDX and XPS analysis the atomic concentration was calculated and presented in Table 1. Interestingly, the concentration in Ni, Zn and Cd calculated on the basis of these methods are different. The atomic concentration from EDX spectra is very close to theoretical values (the concentration of Zn, Mg, Ni and Cd should be equal to 2.9% at.). The atomic concentration from XPS spectra are different. It can be noticed, that the concentration of only Mg is close to the theoretical value. Moreover, the high Zn concentration equal to 9.2% at. was observed. The concentration of Ni and Cd is lower than 1%. However, the XPS spectra, in contrast to EDX spectra collected from TEM, provide information only about the surface composition. Therefore, the conclusion, that the Mg^2+^ ions like Fe^2+^ and Fe^3+^ are uniformly distributed in core and in the surface of materials can be drawn. The Zn^2+^ ions accumulate on the surface of ferrite, whereas Cd^2+^ and Ni^2+^ ions are distributed only in the core of tested material.

**Table 1:** The results of calculations of atomic concentrations on the basis of EDX and XPS spectra in comparison with theoretical values.

chemical element	O	Mg	Fe	Ni	Zn	Cd
theoretical concentration (%)	57.1	2.9	31.3	2.9	2.9	2.9
atomic concentration (EDX) (%)	55.3	2.9	30.9	4.3	3.6	3.0
atomic concentration (XPS) (%)	60.6	2.8	26.0	0.8	9.2	0.6

**Figure 4:**
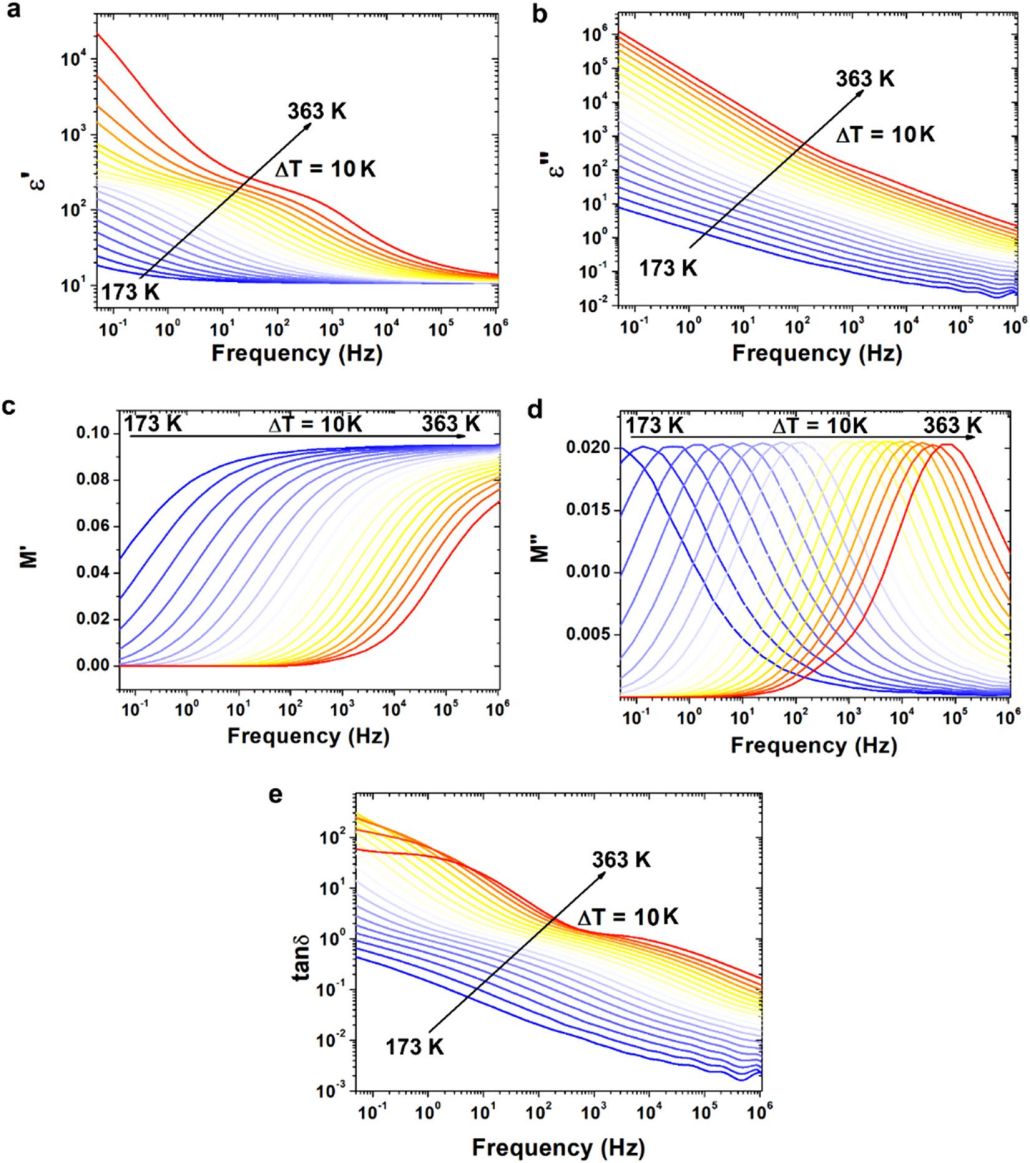
Variation of **(a)** real part of permittivity, **(b)** imaginary part of permittivity, **(c)** real part of electric modulus, **(d)** imaginary part of electric modulus and **(e)** *tanδ* with frequency at wide temperature range 173 to 363 K.

In order to determine dielectric properties of (Zn,Mg,Ni,Fe,Cd)Fe_2_O_4_ high entropy ferrite real and imaginary part of permittivity were measured in the broad temperature range 173–363 K. The dielectric properties of ferrites are strongly associated with temperature, frequency, crystal structure, impurities and humidity^37^. To avoid effect of moisture on the dielectric properties, the sample was firstly heated up to 383 K and kept at this temperature by 10 min. The real (*ε*′) and imaginary (*ε*″) part of permittivity are presented in Fig. 4a,b, respectively. In the *ε*′ diagram, there is a relaxation process visible in the whole measured temperature range. Taking into account the type of material, this relaxation process can be identified as grain boundaries polarization. In the dielectric loss diagram the same process is hardly visible as it is covered by the conductivity. At elevated temperatures, additional electrode polarization phenomenon is visible at low frequencies in the *ε*′ diagram. It is related to the accumulation of charge carriers on the sample-electrode interface. Both processes in low frequency region i.e. electrode and grain boundaries polarizations can be identified as Maxwell-Wagner type polarization^38^. In the low frequency region diffusion or hopping of charges accumulated on grain interfaces requires much more energy than hopping/tunneling in high frequency region^39^. The polarization in ferrites is related to the conduction process discussed later in this study. The electron exchange between Fe^2+^ and Fe^3+^ ions causes local displacement of charge and in consequence the polarization of structure. Moreover, at higher frequencies these movements of charge carriers can lead to formation of small or large polarons^40–42^. The role of grains and grain boundaries in these processes can also be described by analysis of complex electric modulus (M^*^) defined as^43^:

6 $${M}^{\ast }=M^{\prime} +iM^{\prime\prime} =\frac{\varepsilon ^{\prime} }{{|{\varepsilon }^{\ast }|}^{2}}+\frac{i\varepsilon ^{\prime\prime} }{{|{\varepsilon }^{\ast }|}^{2}}$$

where *M*′ and *M*″ are the real and imaginary part of complex electric modulus, respectively.

The electric modulus describes the relaxation of electric field in the materials. The frequency dependent behavior of real and imaginary part of electric modulus can be used to describe electric properties of different materials. The variation of real and imaginary part of electric modulus versus frequency at different temperatures is presented in Fig. 4c,d, respectively. It can be observed that at high temperatures and in the low frequency range the value of *M*′ is equal to 0, which is associated with short-range mobility of charge carriers and electrode polarization. The dispersion of *M*′ value in high frequencies is associated with long-range charge mobility. The conductivity relaxation can be monitored by imaginary part of electric modulus *M*″*(f)* (Fig. 4d). The peaks in this plot are associated with transition between long and short range mobility of charge carriers associated with conductivity by grains and their boundaries^44^. The high frequency range above the maximum of this peak is related to the localized motion of charge carriers within the potential well, whereas low frequency range with conductivity through the grain boundaries.

Additionally, in Fig. 4e dielectric loss tangent (tanδ) defined as:

7 $$tan\delta =\frac{\varepsilon ^{\prime\prime} }{\varepsilon ^{\prime} }$$

has been presented. The value of *tanδ* in low frequency region is very high, which can be associated with leaky capacitive nature of high entropy ferrite. Also in lower frequencies, the high value of dielectric loss can be related to impurities, crystal defects and moisture.

In order to describe electrical conductivity mechanism, complex AC conductivity (σ^*^) was determined on the basis of measured complex permittivity (Eq. 8). The real part of AC conductivity (*σ*_*AC*_) was further analyzed (Eq. 9)

8 $${\sigma }^{\ast }=\omega \varepsilon ^{\prime\prime} {\varepsilon }_{0}+j\omega \varepsilon ^{\prime} {\varepsilon }_{0}$$

9 $${\sigma }_{AC}=\omega \varepsilon ^{\prime\prime} {\varepsilon }_{0}$$

**Figure 5:**
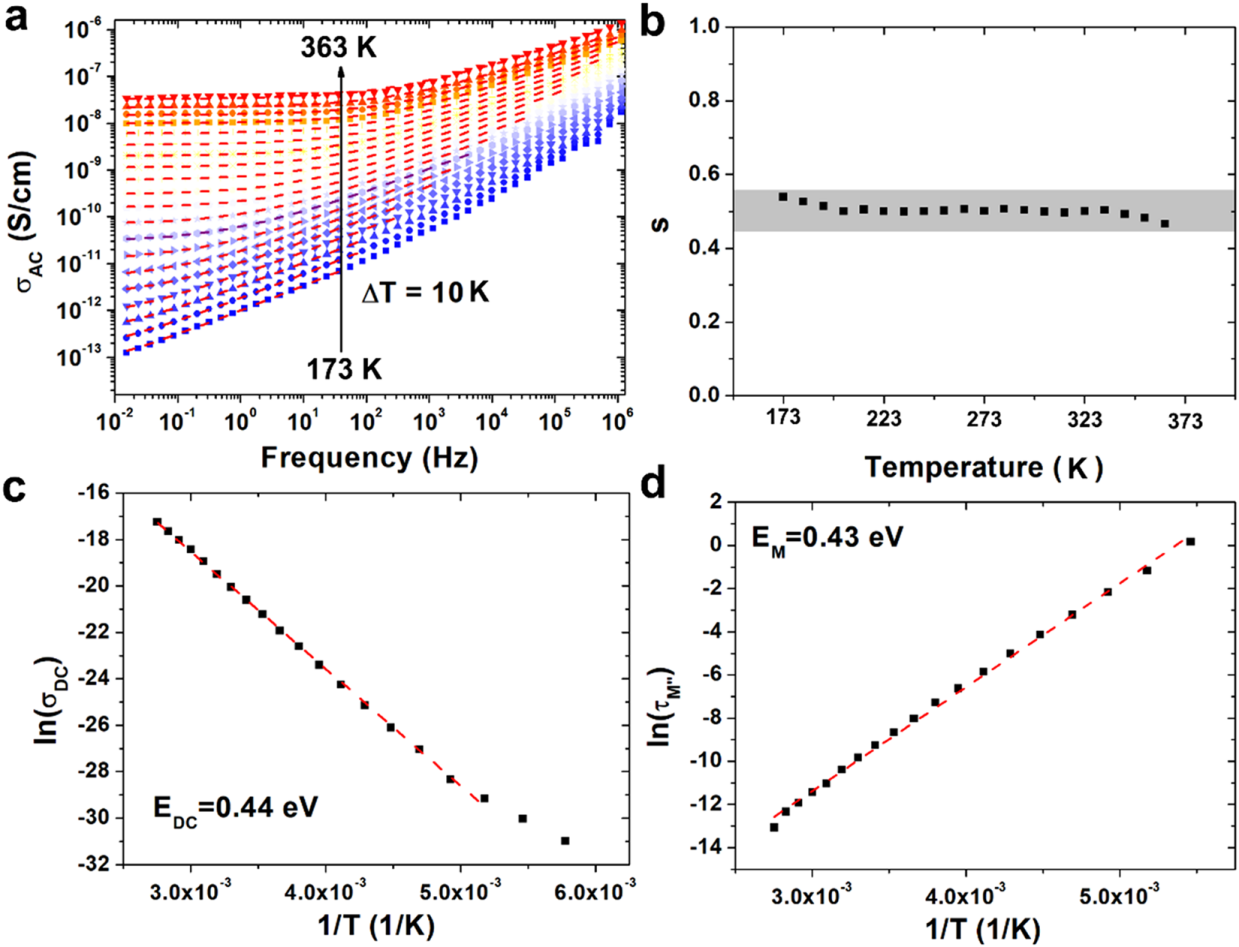
**(a)** AC electrical conductivity measured for compressed sample of (Zn,Mg,Ni,Fe,Cd)Fe_2_O_4_ high entropy ferrite with dashed red lines corresponding to the fit according to Jonscher’s power law, **(b)** *s(T)* behavior with marked area of variation, **(c)** Arrhenius plot (red dashed lines) of DC electrical conductivity associated with conductivity by grain boundaries and **(d)** corresponding to the *M*″ peak associated with transition between long and short range mobility.

Figure 5a illustrates the temperature dependence of *σ*_*AC*_ of (Zn,Mg,Ni,Fe,Cd)Fe_2_O_4_ HEF in a wide range of frequencies. In high frequency region conductivity mechanism is based on hopping or tunneling of charge carriers, while in low frequencies with slow motion of charge carriers. At low frequencies *σ*_*AC*_ is almost constant. This frequency dependence of electrical conductivity can be satisfactorily described by Koop’s phenomenological theory^45^, where conducting grains are surrounded by dielectric grain boundaries^46^. AC electrical conductivity originated from charge hopping can be described by universal power law and expressed as:

10 $${\sigma }_{AC}=A{(2\pi f)}^{s}+{\sigma }_{DC}$$

where *A* is the characteristic parameter, *f* is the frequency, *s* is the exponent dependent on temperature and frequency with values in the range from 0 for ideal ionic-type crystals to 1 for ideal Debye dielectric dipolar-type crystals and *σ*_*DC*_ is the DC electrical conductivity.

In Fig. 5a the best fits were marked by red-dashed lines. Unfortunately, this simple relationship cannot describe AC electrical conductivity in wide frequency range, especially at low temperatures. It can be observed, that at low temperatures *σ*_*AC*_ follows the universal power law only in low frequencies and the maximum limit value of frequency in which universal power law can be used increases with increasing temperature. This behavior in high frequency range can be described by nearly constant-loss (NCL) regime and was observed and described previously in literature^47^. The NCL arises entirely from vibrating ions confined in cages by potential barriers. The *σ*_*DC*_ is associated with diffusion of charge carriers. In higher frequencies Jonscher’s regime occurs and the conductivity is associated with local hopping, while in very high frequencies, unharmonic vibrations might occur^48^. This observation can be successfully used to describe the electrical conductivity in (Zn,Mg,Ni,Fe,Cd)Fe_2_O_4_ HEF. The region without fit presented in Fig. 5a can be related to the NCL, i.e. vibration of ions confined locally in potential wells^49^. In lower frequencies there is a region of Jonscher’s regime, in which electrical conductivity can be characterized by *s(T)* function. The analysis presented in Fig. 5b for (Zn,Mg,Ni,Fe,Cd)Fe_2_O_4_ HEF has been previously used to describe electrical conductivity for different ferrites and magnetite nanoparticles^44,50,51^. It was found that for a high entropy ferrite, exponent *s* is almost independent of temperature, therefore the model of quantum-mechanical tunneling (QMT) of charged carriers between localized states near the fermi level can be applied to describe frequency dependent AC conductivity in analyzed temperature range. The tunneling of charge carriers was observed for the first time by Pollak and Geballe^52^. It was stated previously, that for chalcogenide glasses at low temperatures, QMT model, instead of correlated barrier hopping model has to be applied in order to properly describe *σ*_*AC*_*(f)* relation^53^. The same relationship can occur in high entropy ferrites at low temperatures. In this case this behavior can be associated with difficulties of electron hopping. This electron hopping is associated with existence of Fe^3+^ and Fe^2+^ ions in ferrite structure^54,55^. Hosseinpour *et al*. has stated, that in ferrites the main process of electrical conductivity has two equilibrium reactions^56^. The first one is associated with electron transfer between Fe ions in octahedral site:

11 $${{\rm{Fe}}}^{2+}+{{\rm{Fe}}}^{3+}\leftrightarrow {{\rm{Fe}}}^{3+}+{{\rm{Fe}}}^{2+}$$

The second one is related to the electron hopping between ions of other metals in octahedral site:

12 $${{\rm{Me}}}^{2+}+{{\rm{Fe}}}^{3+}\leftrightarrow {{\rm{Me}}}^{3+}+{{\rm{Fe}}}^{2+}$$

The second reaction causes the change in the initial and final ionic states in the spinel structure, therefore the probability of this electron hopping is negligible in comparison with the first reaction. In (Zn,Mg,Ni,Fe,Cd)Fe_2_O_4_ HEF different ions can occupy octahedral and tetrahedral site. According to the^7,8^ the Fe^2+^, Mg^2+^, Ni^2+^ and Cd^2+^ ions most likely occupies octahedral sites, whereas Zn^2+^ and Fe^3+^ the tetrahedral sites. Moreover, as was presented in Fig. 2b in high entropy ferrite these ions can be randomly distributed, therefore the electron transfer between Fe ions in octahedral site is significantly impeded. The hopping cannot occur and quantum-mechanical tunneling of charged carriers between localized states appears. This, in turn, translates into a presence of NCL regime in wide temperature range and very low electrical conductivity in comparison to other ferrites and magnetite^44^. In low frequencies and at high temperatures the plateau region associated with conductivity by grain boundaries and related to the *σ*_*DC*_ occurs. Increasing conductivity with temperature is related to the increasing electron mobility. The value of DC electrical conductivity can be obtained from the universal power law. Moreover, the activation energy (*E*_*DC*_) of process associated with *σ*_*DC*_ can be calculated according to the Arrhenius law:

13 $${\sigma }_{DC}={\sigma }_{0}\times \exp (\frac{-{E}_{DC}}{{k}_{B}T})$$

where *k*_*B*_ is the Boltzmann constant, *T* is the temperature and *σ*_0_ is the pre- exponential factor. The results of analysis are presented in Fig. 5c. It can be noticed, that the activation energy of this process is equal to 0.44 eV which is similar to *E*_*DC*_ obtained for Ni_0.6_Cu_0.4_Fe_2_O_4_ ferrite (0.43 eV)^57^. Moreover, this value is also similar to the activation energy calculated from relaxation times (Eq. 14) extracted from *M*″ peak (Fig. 5d, 0.43 eV). It was previously described, that the *M*″ peak is associated with transition between long and short range mobility, therefore between conductivity by grain boundaries and grains^44^. The similar value of activation energy confirms that observation for HEF.

14 $$\tau ={\tau }_{0}\times \exp (\frac{{E}_{M}}{{k}_{B}T})$$

**Figure 6:**
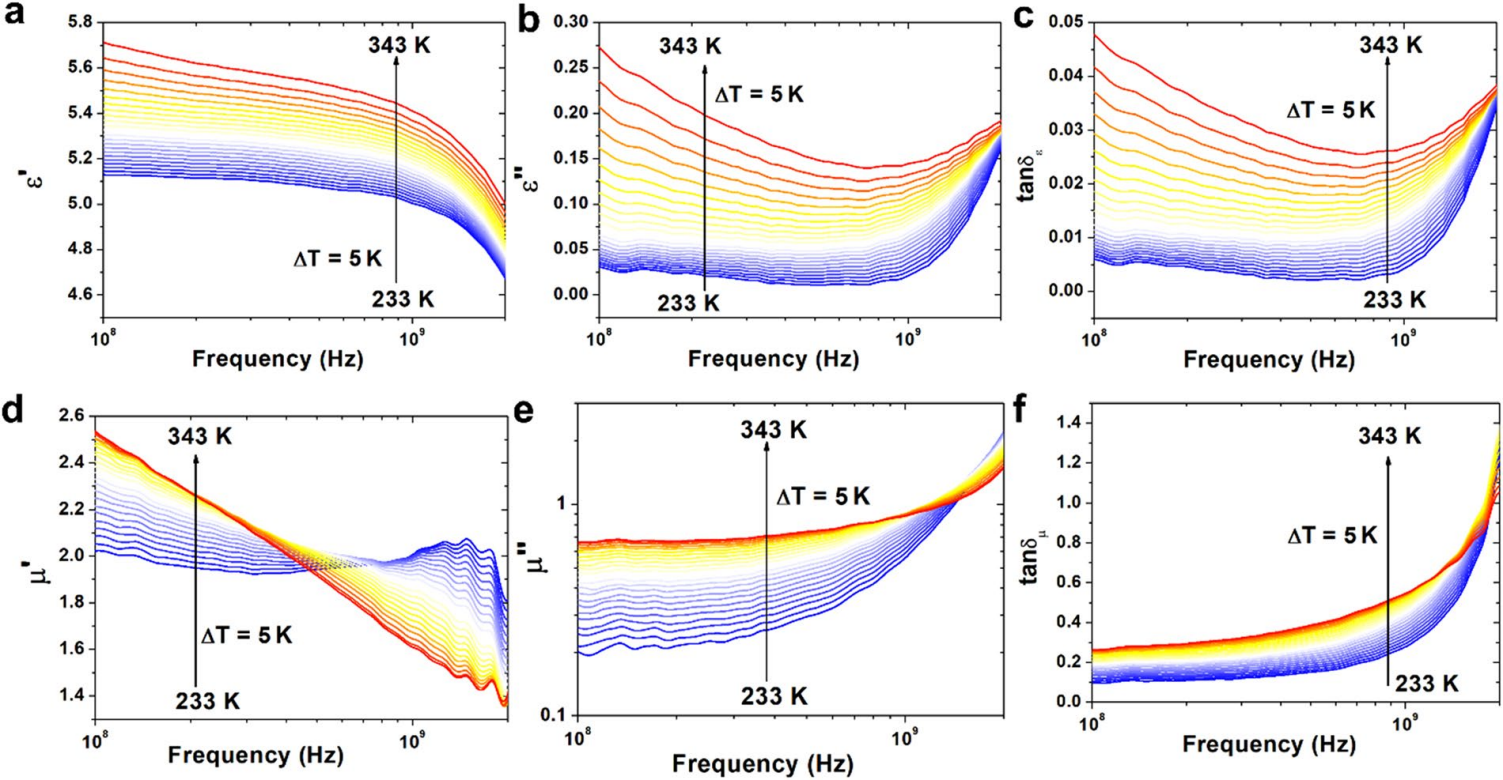
The high frequency dielectric and magnetic properties of (Zn,Mg,Ni,Fe,Cd)Fe_2_O_4_ high entropy ferrite at wide temperature range: **(a)** real part of permittivity, **(b)** imaginary part of permittivity, **(c)** *tanδ*_*ε*_, **(d)** real part of permeability, **(e)** imaginary part of permeability, **(f)** *tanδ*_*μ*_.

The ferrites and nanoferrites can find application in high frequency range, for example as RF and microwave filters, microwave absorbers and antenna magneto-dielectric substrates. This is associated with their dielectric and magnetic properties. In order to determine the possibility of using of (Zn,Mg,Ni,Fe,Cd)Fe_2_O_4_ high entropy ferrite in these applications their complex permittivity and complex permeability in high radio frequency range were measured and presented in Fig. 6. Moreover, the influence of temperature on permittivity and permeability was determined at wide temperature range. Figure 6a,b presents changes in the value of real and imaginary part of permittivity with increasing temperature. Permittivity of ferrites in high frequencies is depending on the electronic and atomic polarization of grains and polarization of defects^58^. The nearly constant values of ε′ in wide frequency range can be associated with the fact that the interfacial and dipolar polarizations play important role at lower frequencies (according to the Koop’s theory), whereas in higher frequencies, especially in GHz region, changes (observed for (Zn,Mg,Ni,Fe,Cd)Fe_2_O_4_ HEF) can be associated with polarization of defects. Moreover, the changes in real part of permittivity with increasing temperature are minor (for 100 MHz 5.12 and 5.71 at 233 and 343 K, respectively). The changes in the value of dielectric losses (imaginary part of permittivity) are more noticeable. However in higher frequencies the value of *ε*″ is weakly dependent on temperature, which can be associated with polarization of defects presented in structure of high entropy ferrite. The same relationship is visible for the loss tangent *tanδ*_*ε*_ (Fig. 6c). The presented in Fig. 6d,e results confirm, that also complex permeability is strongly temperature dependent. This is important to determine further application of different magneto-dielectric materials in low and high temperature region. For example, at 100 MHz the value of real part of permeability changes from 2.02 to 2.54 in 233–243 K temperature range, whereas imaginary part of permeability increases from 0.2 to 0.66. However these changes are not linear and different for low and high frequency region. It can be observed, that in higher frequencies real and imaginary parts of permeability decrease with increasing temperature. The initial permeability at lower frequencies is strongly dependent on the chemical composition, temperature and microstructure. The low value of real part of permeability indicate a single domain structure, therefore the domain wall motion is absent and initial permeability is low and associated only with spin rotation (grains are too small to establish an intra-granular domain walls inside the grain). The low value of real part of permeability and high value of magnetic losses can be then associated with grain boundaries, small particle size and chemical inhomogeneity of (Zn,Mg,Ni,Fe,Cd)Fe_2_O_4_ HEF. The high value of *tanδ*_*μ*_ is associated with nanostructure of high entropy ferrite and indicates the possibility of using (Zn,Mg,Ni,Fe,Cd)Fe_2_O_4_ HEF as absorber of RF and microwave radiation.

To determine electromagnetic interference shielding properties two key factors: reflection loss (*RL*) and shielding effectiveness (*SE*) were calculated in wide frequency and temperature range^59,60^

15 $$RL=20log|\Gamma |$$

16 $$SE=-\,20\,\log |\frac{(1-{\Gamma }^{2})T}{1-{T}^{2}{\Gamma }^{2}}|$$

17 $$\Gamma =\frac{\sqrt{\frac{(\mu ^{\prime} -j\mu ^{\prime\prime} )}{(\varepsilon ^{\prime} -j\varepsilon \text{'}\text{'})}}\,\tanh (j\frac{2\pi fd}{c}\sqrt{(\mu ^{\prime} -j\mu ^{\prime\prime} )(\varepsilon ^{\prime} -j\varepsilon ^{\prime\prime} )})-1}{\sqrt{\frac{(\mu ^{\prime} -j\mu ^{\prime\prime} )}{(\varepsilon ^{\prime} -j\varepsilon ^{\prime\prime} )}}\,\tanh (j\frac{2\pi fd}{c}\sqrt{(\mu ^{\prime} -j\mu ^{\prime\prime} )(\varepsilon ^{\prime} -j\varepsilon ^{\prime\prime} )})+1}$$

18 $$T={e}^{-(j\frac{2\pi fd}{c}\sqrt{(\mu ^{\prime} -j\mu ^{\prime\prime} )(\varepsilon ^{\prime} -j\varepsilon ^{\prime\prime} )})}$$

where *Γ* is the reflection coefficient, *T* is the transmission coefficient, *ε*′ and *ε*″ are the real and imaginary parts of complex electric permittivity, *μ*′ and *μ*″ are the real and imaginary parts of complex magnetic permeability, *f* is the frequency of electromagnetic radiation, *c* is the speed of light and *d* is the absorber thickness.

**Figure 7:**
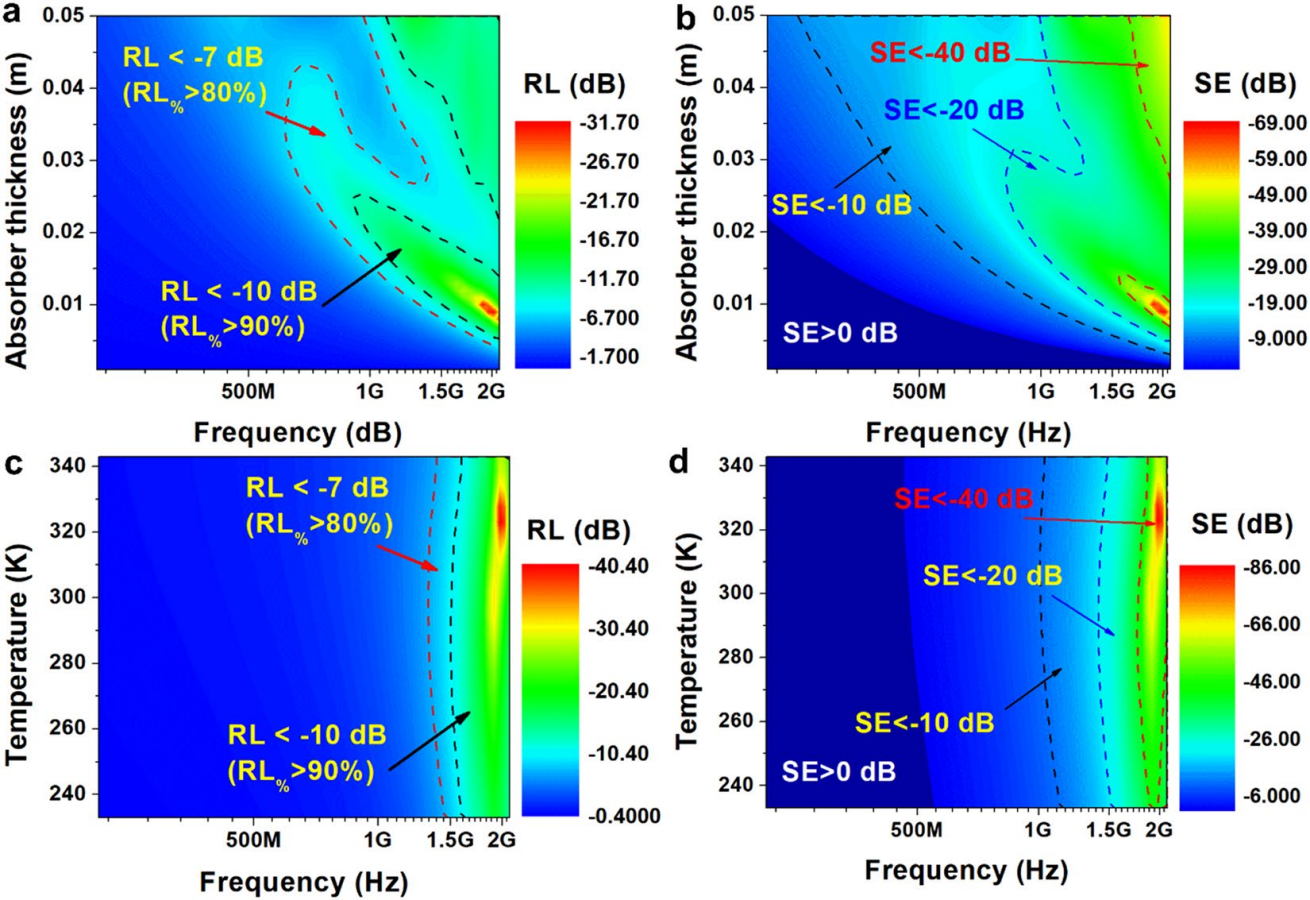
The influence of absorber thickness on the reflection loss **(a)** and shielding effectiveness **(b)** calculated for constant temperature (*T* = 278 K); temperature dependent reflection loss **(c)** and shielding effectiveness **(d)** calculated for (Zn,Mg,Ni,Fe,Cd)Fe_2_O_4_ HEF with *d* = 1 cm.

The analysis was performed for different absorber thicknesses (*d*) (from 1 mm to 5 cm) and temperatures (from 233 to 343 K). The analysis results are presented in Fig. 7. Figure 7a,b present influence of absorber thickness on the reflection loss and shielding efficiency with marked two areas corresponding to the *RL* > 80% and *RL* > 90%. The area with *RL* > 90% is the effective bandwidth, in which *RL* < −10 dB and correspond to the effective absorber thickness i.e. thickness of layer of (Zn,Mg,Ni,Fe,Cd)Fe_2_O_4_ HEF, which can be used to absorb RF and microwave radiation. As can be seen in Fig. 7b in the same area *SE* is negative, therefore interaction of electromagnetic wave with material results in the absorption and dissipation of that energy as heat. The *RL* < −10 dB for all tested absorber thicknesses can be observed mostly in GHz region. For the absorber thickness in the range from 2 to 2.5 cm the same RL can be achieved even at 0.9 GHz. It is well known fact, that the absorption of RF radiation is more difficult than of microwave radiation, but equally important. Therefore *RL* < −7dB was also marked in the same 2D contour plot. As can be seen in Fig. 7a the *RL* < −7dB contour line reaches 600 MHz frequency, however minimal thickness of absorber is in this case 3.3 cm. At the same frequency region *SE* is lower than −10 dB, therefore *RL* is associated with absorption of RF radiation. The analysis of Fig. 7a,b allows to state, that the highest microwave absorption properties can be obtained for layer with thickness from 1 to 0.8 cm at 1.9 to 2.1 GHz, respectively. For this range *RL* < −25 dB and *SE* < −50 dB. The temperature dependence on the electromagnetic interference shielding properties has not been well described in the literature, however temperature is a very important parameter affecting the dielectric and magnetic properties of materials. The temperature dependent EMI shielding properties were described for Ti_3_AlC_2_ ceramics, graphene nanosheet reinforced alumina ceramics and CVI-SiC_f_/BN/SiC composites^61–63^. Figure 7c,d show 2D contour plots representing the influence of temperature on the EMI shielding properties of (Zn,Mg,Ni,Fe,Cd)Fe_2_O_4_ HEF. As can be seen the influence of temperature on the frequency range in which tested material can be characterized by good absorption properties is minor. For example *RL* < −10 dB can be observed from 1.5 to 1.6 GHz in the range from 233 K to 343 K. The most significant differences can be seen in the peak value of *RL* (*RL*_*min*_). The peak value of *RL* is observed close to 1.9 GHz for studied temperature range. By comparison of *RL* for f = 1.9 GHz and d = 1 cm following values have been obtained: *RL* = −25.6 dB at 278 K, *RL* = −18 dB at 233 K, *RL* = –31.2 dB at 298 K and *RL* = −19.4 dB at 343 K. As one can see the impact of temperature on peak value of RL is significant. The similar results can be observed for shielding effectiveness. For the same absorber parameters (f, d) and at the same temperatures (i.e. 233, 278, 298 and 343 K) *SE* is equal to −40.7 dB, −56.3 dB, −67.5 dB, −43.1 dB, respectively. The presented results indicate that the (Zn,Mg,Ni,Fe,Cd)Fe_2_O_4_ HEF can be used as microwave absorber in L and S-band and even at lower frequencies of UHF band.

## Conclusions

In this study synthesis, structure and properties of (Zn,Mg,Ni,Fe,Cd)Fe_2_O_4_ high entropy ferrite were described. The high entropy oxides are new group of materials with unique electric and dielectric properties. The new method of synthesis of high entropy ferrite, which was proposed in present work, allowed us to synthesize nanostructure material with average crystallite size equal to 11.8 nm and random distribution of different ions, which was confirmed by analysis of STEM-EDX 2D maps. Additionally, the analysis of XPS spectra can allow to state, that Zn^2+^ ions accumulate on the surface of HEF. The dielectric and electrical properties, as well as interference shielding properties of (Zn,Mg,Ni,Fe,Cd)Fe_2_O_4_ high entropy ferrite were described in detail. Electrical polarization in (Zn,Mg,Ni,Fe,Cd)Fe_2_O_4_ high entropy ferrite was related to the electron transfer between Fe^2+^ and Fe^3+^ ions and the dispersion of real part of permittivity and real part of electric modulus were associated with two different mechanisms: short- and long-range mobility of charge carriers. It was noted, that for high entropy ferrite with random distribution of different ions in tetrahedral site electron hopping cannot occur and quantum-mechanical tunneling of charged carriers between localized states appears, which is manifested by low electrical conductivity. The observed short-range mobility in high frequencies was related to the tunneling of electrons between Fe^2+^ and Fe^3+^ ions whereas the long-range mobility was associated with diffusion of charge carriers through grain boundaries. The AC electrical conductivity was described by three different regimes: DC electrical conductivity in low frequencies, Jonscher’s regime in high frequency region and nearly-constant loss arising entirely from vibrating ions confined in potential barrier cages in highest frequencies. The activation energy of DC electrical conductivity for (Zn,Mg,Ni,Fe,Cd)Fe_2_O_4_ high entropy ferrite was equal to 0.43 eV and it was similar to activation energy determined from *M*″ peak (0.44 eV). The high frequency complex permittivity and permeability were strongly temperature dependent. The peak value of RL i.e. *RL*_*min*_ depends strongly on temperature. For example, absorber with *d* = 1 cm at *f* = 1.9 GHz has *RL* = −25.6 dB at *T* = 278 K, while *RL* = −18 dB at *T* = 233 K, *RL* = −31.2 dB at *T* = 298 K and *RL* = −19.4 dB at *T* = 343 K. The highest microwave absorption properties can be obtained for layer with thickness from 1 to 0.8 cm at 1.9 to 2.1 GHz, respectively. For this range *RL* < −25 dB and *SE* < −50 dB. The low value of real part of permeability and high value of magnetic losses at high frequency region can be associated with grain boundaries, small particle size and chemical inhomogeneity.
